# Assessing for domestic violence in sexual health environments: a qualitative study

**DOI:** 10.1136/sextrans-2017-053322

**Published:** 2017-08-04

**Authors:** Jeremy Horwood, Andrew Morden, Jayne E Bailey, Neha Pathak, Gene Feder

**Affiliations:** 1 The National Institute for Health Research Collaboration for Leadership in Applied Health Research and Care West (NIHR CLAHRC West) at University Hospitals Bristol NHS Foundation Trust, Bristol, UK; 2 School of Social and Community Medicine, University of Bristol, Bristol, UK; 3 Women’s Health Research Unit, Queen Mary University of London, London, UK

**Keywords:** Clinical Care (general), Compex Interventions, Qualitative Research, Sexual Abuse

## Abstract

**Objectives:**

Domestic violence and abuse (DVA) is a major clinical challenge and public health issue. Sexual health services are an important potential site of DVA intervention. The *Assessing for Domestic Violence in Sexual Health Environments* (ADViSE) intervention aimed to improve identification and management of DVA in sexual healthcare settings and is a modified version of the *Identification and Referral to Improve Safety* (IRIS) general practice programme. Our qualitative evaluation aimed to explore the experiences of staff participating in an IRIS ADViSE pilot.

**Methods:**

Interviews were conducted with 17 sexual health clinic staff and DVA advocate workers. Interviews were audio recorded, transcribed, anonymised and analysed thematically.

**Results:**

Staff prioritised enquiring about DVA and tailored their style of enquiry to the perceived characteristics of patients, current workload and individual clinical judgements. Responding to disclosures of abuse was divided between perceived low-risk cases (with quick onwards referral) and high-risk cases (requiring deployment of institution safeguarding procedures), which were viewed as time consuming and could create tensions with patients. Ongoing training and feedback, commissioner recognition, adequate service-level agreements and reimbursements are required to ensure sustainability and wider implementation of IRIS ADViSE.

**Conclusions:**

Challenges of delivering and sustaining IRIS ADViSE included the varied styles of enquiry, as well as tensions and additional time pressure arising from disclosure of abuse. These can be overcome by modifying initial training, providing regular updates and stronger recognition (and resources) at policy and commissioning levels.

## Background

Domestic violence and abuse (DVA), a violation of human rights, is a major clinical challenge and public health issue with far-reaching and devastating consequences for individuals, families and society.^1 2^ In the UK, the National Health Service (NHS) can be the first point of contact for people experiencing DVA and plays a major role in dealing with the impact of DVA.^3^ The estimated cost of DVA to the NHS is £1.7 billion per year.^4^

The UK National Institute for Health and Care Excellence (NICE) guidelines for DVA recommend that front-line staff in all health and social care services are trained to be aware of the indicators of DVA and recommend directly asking patients about abuse.^3^ For some settings, including sexual health services, NICE judged that asking all patients ‘should be a routine part of good clinical practice’. Health services can be crucial in responding to and helping prevent further DVA by intervening early, providing treatment and information and referring patients to specialist DVA services.^5 6^ The BASHH has recently published guidance for responding to DVA in sexual health services.^7^ These services are well placed for DVA intervention^8^ because of increased unintended pregnancies^9 10^ and sexual health problems in people who experience DVA or associated physical or sexual violence.^11–13^

Systematic interventions to promote DVA enquiry and management in sexual health settings are rare. Bacchus and colleagues’ intervention improved enquiry rates and support for women, but did not universally engender routine enquiry (or the confidence to do so) and could potentially cause harm (such as negative labelling of patients by professionals, breaches  of confidentiality or failing to act on information appropriately).^8^ The *Identification and Referral to Improve Safety* (IRIS) programme (http://www.irisdomesticviolence.org.uk/iris/ 6) is an evidence-based training intervention for general practice staff to identify, respond and refer appropriately women experiencing DVA. IRIS was shown to be effective,^6^ cost effective^14^ and to improve clinicians’ knowledge of DVA and perception of role in routine clinical enquiry for DVA.^15^ IRIS is now being implemented nationwide in general practice. IRIS *Assessing for Domestic Violence in Sexual Health Environments* (ADViSE) is an adaptive pilot study to investigate the feasibility and initial effectiveness of an IRIS-based training programme for sexual health staff, aiming to increase enquiry about (and disclosure of) DVA, improve the professional response to disclosure and increase referral to specialist DVA advocacy services. IRIS ADViSE^16^ was a complex intervention featuring a modified IRIS training package for all staff within a sexual health clinic (clinical staff had an initial 2-hour session and 1-hour follow-up, reception staff had a 1-hour session), updates to existing electronic patient record (EPR) templates (including a mandatory prompt for clinical staff to ask about DVA), provision of local referral pathways to local DVA services providing advocacy and use of supporting materials such as posters, information sheets and discrete cards highlighting the DVA advocacy service to patients and referral algorithms for staff. Details of the development and piloting of IRIS ADViSE, including achievement of feasibility outcomes, are reported separately.^17^ The aims of this study were to explore the perceptions and experiences of sexual health clinic staff and DVA advocates after participation in the IRIS ADViSE pilot and to investigate factors which may influence implementation and outcomes.

## Methods

### Data collection

In-depth semistructured interviews were conducted to investigate professionals’ views on training received; their confidence, knowledge and ability to deliver IRIS ADViSE; barriers and facilitators to delivery; perceived impact for patients and their own clinical practice; and sustainability. Ethical approval was obtained from University of Bristol Faculty of Medicine and Dentistry Research Ethics Committee (FREC 19022). At interview, participants were asked for written informed consent, following which a flexible topic guide was used.

### Recruitment and sampling

A purposive sample was drawn in relation to staff role (receptionist, health adviser, nurse manager, nurse, ‎doctor, registrar, consultant and DVA advocate) in order to capture the views of a range of staff who had participated in IRIS ADViSE. An invitation email and study information sheet was issued to clinical staff and DVA advocates and those interested in taking part were asked to contact AM. Interviews were analysed in batches, and sampling continued until no new themes emerged from the interviews by the end of data collection.^18^ A total of 17 interviews were undertaken, lasting between 30 min and 1 hour.

### Analysis

All interview were audio-recorded, transcribed, anonymised, checked for accuracy and then imported into QSR NVIVO V.10 qualitative data analysis software. Data were scrutinised using data-driven inductive thematic analysis^19^ to identify and analyse patterns and themes. An initial coding frame was developed by AM and new data compared with previous data and refined if necessary. Codes were built into broader categories through comparison across transcripts and higher-level recurring themes were developed. A subset of transcripts were independently scrutinised against emergent coding frames by a senior social scientist (JH) and discrepancies were discussed to contribute to the generation and refinement of codes to maximise rigour.^19^ Emergent themes were discussed by the multidisciplinary research team (JH, AM, JEB, NP and GF) to ensure credibility.

## Results

The sample consisted of 17 doctors, allied healthcare professionals, reception staff and DVA advocates (see table 1). Participants ranged in level of clinical or DVA support experience and the majority were female. Analysis led to the development of key emergent themes: putting DVA enquiry into practice, patient responses to DVA enquiry, handling disclosures, impact on clinical practice, and sustainability facilitators and barriers.

**Table 1:** Demographic characteristics of study participants (n=17)

Staff group	Total	Female	Experience (years)				Participant pseudonyms
			0–4	5–9	10–15	15+	
Medical * (consultants, junior doctors and specialty trainees)	5	5	0	1	2	2	Mrs A Mrs B Mrs C Mrs G Mrs L
Front-line* (nurse managers, nurses, health advisers, receptionists)	9	6	2	4	1	2	Mrs D Mr E Mr F Mrs I Mrs J Mr K Mrs M Mrs O Mrs Q
DVA advocator	3	3	2	1	0	0	Mrs H Mrs N Mrs P

*Aggregated groups to ensure participant anonymity.

### Putting DVA enquiry into practice

A prompt to ask a question about DVA was embedded within the clinic’s EPR system. While in some instances it could act as an irritant for clinicians or patients (especially in relation to follow-up appointments), the addition to the template was widely seen as a positive tool for prompting enquiry about DVA, compelling clinicians to ask the question:

*Because it [DVA prompt] was integrated into the proforma, you don’t really … well, you do have a choice, cause you can just tick that you haven’t asked … I’m the type of person who would feel guilty or that I’d missed something if I hadn’t asked, so I do try and ask most people. (Mrs C)*

The majority of clinic staff described how, after receiving the IRIS ADViSE training, they felt knowledgeable and confident about asking patients about DVA in practice. For a few clinicians, some initial concerns were discussed including identifying perpetrators, handling a ‘real-life case’ and causing offence by enquiring. The most common was the challenge of introducing a sensitive question into the flow of the consultation:

*If I’m gonna bring up something difficult, then I’ll preface it with something. If I’m gonna ask them about their sexual history I’ll say, ‘I need to ask you some more personal questions. Is that okay?’—or something like that. So I’m quite used to this sort of thing, but I found it hard to work out where to slot it in and how to introduce the question. (Mrs A)*

Clinicians worked to modify their approach and this was apparent in four different ways of asking about DVA which often overlapped. First, some clinicians felt that the most comfortable way of asking was emphasising the routine nature of the enquiry and closely following the direct wording of the EPR proforma:

*I think my strategy really is to, you know, I’ll explain to people we’ve got lots of things that we ask people. We ask everybody the same questions. We’re not, you know, I make it quite plain that I’m not asking them for any particular reason. (Mrs J)*

Second, other clinicians thought that rephrasing or ‘softening’ the question in order to prevent causing offence was more appropriate, for example, by asking ‘if everything is alright at home’ rather than asking directly about DVA. Third, some clinicians asked more targeted questions of those patients who they perceived as ‘at risk’:

*If they say ‘no’ and I’ve a very strong suspicion I would be willing to ask more questions. Yeah that’s the other thing you pick it up you see, and where you’d look for it before you, you know, you’d be knowing that that could be a … you’re making it that that’s definitely on your radar. Yeah. Your vulnerable adult you’d be even more worried about. (Mrs M)*

Fourth, and relatedly, was the obverse process of deprioritising individuals deemed unlikely candidates of abuse, or in order to manage time constraints within consultations:

*I have to admit, being honest, that there are times when I’m doing something like a complex coil fit, which is a very long, complicated appointment, which you don’t really have enough time to do anyway, that there are ways to ask the question to get a negative answer if you’re in a hurry. (Mrs B)*

### Patient responses to DVA enquiry

The EPR prompt meant that both men and women were asked about DVA. Clinicians described common gendered reactions to enquiry, highlighting a perception that women were more likely to disclose and men (while occasionally disclosing) would either be surprised and annoyed or, more usually, humorously made light of the question.

Notwithstanding some patients who were annoyed or surprised at being asked, most were described as reacting well, were glad to have the opportunity to disclose (even if they did not) and thought it was a positive thing that they were asked:

*… they have welcomed being asked about it, even if they’ve not ever been involved in an incident of domestic abuse themselves, that they appreciate that people are asking that question. (Mrs L)*

### Handling disclosures

Clinic staff stated that they felt prepared and happy to handle perceived ‘low-risk’ cases which they defined as either patients who could (a) be easily referred to the DVA partner agency or (b) who could be provided with information (written or verbal) to support their decision about how to proceed. Clinic staff described their biggest challenge was encountering perceived ‘high-risk’ cases featuring children and the need to instigate associated safeguarding procedures:

*… children involved then they start to go ‘Oh hang on a minute, why are you asking me about my children’ and then they get worried … they try and reassure you that it never happens in front of the kids or you know I am sure it doesn’t, but it’s just that how do you know and it’s and it puts us in a tricky position sometimes … this is where it can take all the time up. (Mrs G)*

Another significant challenge was the problem presented by the lack of resources for the management of perpetrators (male or female) and male victims:

*I mean, there’s very little, isn’t there, support for men who are kind of victims of abuse or violence … but I think sometimes it’s just, you know, being honest with people and kind of saying that. (Mr F)*

### Impact on clinical practice

Reflecting on the impact of the IRIS ADViSE intervention, all participants maintained the view that routine enquiry and partnership working for referral of DVA was important and fitted with the remit of sexual health services, that participating in the training and delivery of the programme had raised awareness and focus regarding DVA, and improved the ability of clinical staff to detect and manage instances of DVA:

*I think the positive side of that would be that they are asked, and I think it’s important to do that, and it’s definitely an area that I think people probably will come to who might not disclose at other places, and that means that you can help people who need that support. (Mr E)*

Notwithstanding these benefits, there was a cost for clinic staff: namely eating into the time available to deal with patients. The consequence impacted on two levels. First, in terms of meeting service level agreements:

*We’ve got to see specific numbers of people … And so there’s pressure from the Commissioners and from the [NHS] Trust, that we have got to see a certain number of people and as the requirements get bigger, then we are obviously going to need to see less patients. (Mrs G)*

Second, by adding to the workloads of clinic staff in a context in which they increasingly are requested to ask contextual questions (such as alcohol and substance use) which entail additional administrative burdens:

*If we’re going to be asking about things like domestic violence, cos we’re also being encouraged to ask about drug abuse, alcohol use, risky sex, etc, you know, the list of things that we’re asking about seems to be getting longer, whereas the time allocated to each seems to be getting shorter so there has to be some I think agreement about, you know, where those two things are going to meet, some balance between the two. (Mrs L)*

### Sustainability facilitators and barriers

Participants suggested an important facilitating strategy to ensure continuation of the IRIS ADViSE approach would be to provide ongoing feedback and training. This included annual full training for new staff, with less frequent ‘refresher’ sessions with feedback on disclosure rates and further discussion. A second need identified by participants is for strong internal leadership and key points of contact to cumulatively drive the initiative forward, maintain positivity and deal with operational queries:

*From the point of view of the actual referral process, knowing that there is generally somebody available … within our clinic we’re lucky because we’ve got people who are resources within the clinic that we can always bounce things off. (Mr K)*

The final factor revolved around the interaction between policy-level support and provision of service-level agreements from local NHS Trusts and commissioning groups to ensure that financial reimbursement was provided, time allowances were made and support and recognition of the service given:

*Mrs G: Everyone’s feeling pressure at the moment you know, I think if they can get, if you can get the Commissioners involved. You know to say that they would like it done and it would be in part of their SLA, you know the agreement …*

*R: Service Level Agreement, yeah?*

*Mrs G: Yeah, you know, if they because you know we are happy to do it, it’s just sort of telling the Trust, recognising that this takes time, so we sort of often agree to do things, then it will have an impact on the service.*

This concern was shared by the DVA advocates, who identified the need to be resourced to provide provision of their services linked to interventions:

*I think obviously it’s sustainable if you have the staff and, you know, managing the caseload obviously there’s funding cuts which you need to worry about. (Mrs P)*

## Discussion

The IRIS ADViSE intervention provided an explicit process for responding to DVA in a sexual health setting. A separate quantitative evaluation of the IRIS ADViSE pilot found that over 3 months the DVA enquiry rate was 61% (n=1090, the DVA identification rate was 7% (n=79) and 10% (8 out of 79) of patients were referred to a DVA advocate).^17^ Even though DVA enquiry was not routinely asked to all patients, it demonstrated a marked improvement in relation to the three months preceding the intervention pilot where no cases of DVA identified or referrals to DVA advocates were recorded.

Our findings suggest that having a mandatory question embedded within an EPR was an aid to enquiry^20^ and participants described feeling confident and prepared after the training, consistent with the experiences of general practice staff who received the original IRIS training.^15^ Conversely staff recounted difficulties in ensuring a ‘comfortable’ consultation for patients and managing the additional time pressure arising from disclosure of abuse^8 21^ when putting enquiry into practice. These factors influenced clinicians’ style and selective approach to enquiry, often based on intuition, the patient’s presentation and risk markers.^21–23^ A varying approach to DVA enquiry, selecting some patients to ask and different ways of asking about DVA to managing likelihood of disclosure, demonstrates the inherent tension of introducing routine DVA enquiry, while also allowing staff to use their clinical acumen to make the enquiry appropriate to each situation. Participants highlighted the need for ongoing training and feedback on disclosure rates to aid staff performance and delivery of the intervention.^21^ Future implementation of the intervention could consider expanding the role-play aspect of the training to improve staff confidence in how to introduce DVA questions within the consultation and how to best handle DVA disclosure involving children.

Participants reported that ‘low-risk’ disclosures, when patients could be easily referred to the partner DVA advocate service or provided with information, were considered relatively simple and easy to handle, consistent with the preference of general practice clinicians for an identification and referral role managing DVA.^15^ However, cases with an immediate risk of harm to the patient or their children were more complex in terms of managing the patient’s wishes and navigating existing safeguarding procedures which added an increased workload and administrative burden.^24^ This tension of managing patient confidentiality alongside safety of children echoes the experiences of general practice staff.^25^ Our data also highlight that adequate referral options and support resources for perpetrators and male victims were not available.^8 26^ This links to a broader problem stemming from uncertainty about effective interventions to address perpetration of DVA^5^ and the challenges of devising services that male victims will engage with.^23^

Our study is a novel exploration of implementing a complex DVA intervention in a sexual health service. Although the participant sample was drawn from a single sexual health clinic and third-sector organisation, a diverse sample of participants in terms of professional roles was interviewed and enabled a comprehensive insight into IRIS ADViSE from multiple perspectives, with analysis showing commonality in views and experiences. Achievement of data saturation,^18^ together with the rigour of analysis, improves the credibility of findings.

The recent UK Government Violence against Women and Girls Strategy^27^ recognises the IRIS model as an effective programme for general practice and promotes the need to make early detection and prevention of DVA a priority for services, such as sexual health clinics, where the opportunity for early intervention is possible. Although recent BASHH guidelines have been produced to support sexual health services who want to introduce DVA enquiry,^7^ our findings highlight the inherent tension in DVA enquiry which, while viewed as an important and useful intervention that fits with the remit of sexual health services,^21 28^ is set against the potential for increased workload and implications for time (individually and for the clinic). This is consistent with research in other healthcare settings which have implemented DVA screening approaches.^20 21 29^ If resources were available, the intervention could be improved by developing internal pathways to pass DVA cases to other staff members (eg, health advisers) when time pressures preclude clinical staff from adequately responding to DVA. Findings from previous DVA screening interventions report the importance of interventions being congruent with existing policy-driven demands placed on clinicians.^30^ In this context, recent UK policy initiatives to enquire about lifestyle (such as alcohol and substance use) routinely in consultations^31^ can have an adverse effect on also being able to additionally enquire about DVA in time-limited consultations. This is ironic, given the association between substance abuse and DVA. Our findings identify the need to formally incorporate DVA training programmes into agreements with local trusts and commissioners to ensure ongoing commitment and support in the form of funding, service-level agreements and priorities. The funding needs to include linkage with a local DVA service that can deliver training and establish a referral pathway.

**Key messages**

- All participants felt that the enquiry and referral for domestic violence and abuse (DVA) was appropriate and valuable in sexual health settings.
- Participants identified barriers including fitting enquiry into variable clinical contexts and managing additional time and workload demands from more complex referrals.
- Long-term commitment and engagement from commissioners and local trusts is required to support DVA training and support programmes for enquiry and referral pathways.

## References

[1] Ellsberg M, Jansen HA, Heise L, et al Intimate partner violence and women’s physical and mental health in the WHO multi-country study on women’s health and domestic violence: an observational study. Lancet 2008;371:1165–72. 10.1016/S0140-6736(08)60522-X 18395577

[2] Garcia-Moreno C, Jansen HA, Ellsberg M, et al Prevalence of intimate partner violence: findings from the WHO multi-country study on women’s health and domestic violence. Lancet 2006;368:1260–9. 10.1016/S0140-6736(06)69523-8 17027732

[3] National Institute for Health and Care Excellence. Domestic violence and abuse: multi-agency working. NICE guideline: London, 2014.

[4] Walby S The cost of domestic violence: up-date. Lancaster: Lancaster University, 2009.

[5] National Institute for Health and Care Excellence. Domestic violence and abuse: how health services, social care and the organisations they work with can respond effectively. London: National Institute for Health and Care Excellence, 2014.

[6] Feder G, Davies RA, Baird K, et al Identification and referral to improve safety (IRIS) of women experiencing domestic violence with a primary care training and support programme: a cluster randomised controlled trial. Lancet 2011;378:1788–95. 10.1016/S0140-6736(11)61179-3 22000683

[7] Sacks R, Dhairyawan R, Brawley D, et al Responding to domestic abuse in sexual health settings. London: British Association for Sexual Health and HIV (BASHH) Sexual Violence Group, 2016.

[8] Bacchus LJ, Bewley S, Vitolas CT, et al Evaluation of a domestic violence intervention in the maternity and sexual health services of a UK hospital. Reprod Health Matters 2010;18:147–57. 10.1016/S0968-8080(10)36526-8 21111359

[9] Miller E, McCauley HL, Tancredi DJ, et al Recent reproductive coercion and unintended pregnancy among female family planning clients. Contraception 2014;89:122–8. 10.1016/j.contraception.2013.10.011 24331859 PMC4018410

[10] Öberg M, Stenson K, Skalkidou A, et al Prevalence of intimate partner violence among women seeking termination of pregnancy compared to women seeking contraceptive counseling. Acta Obstet Gynecol Scand 2014;93:45–51. 10.1111/aogs.12279 24117134

[11] Sanmani L, Sheppard ZA, Chapman C Factors associated with the anonymous reporting of lifetime domestic violence in a genitourinary medicine clinic: a patient self-reported questionnaire study. Int J STD AIDS 2013;24:401–7. 10.1177/0956462412472799 23970710

[12] El-Bassel N, Gilbert L, Wu E, et al Intimate partner violence prevalence and HIV risks among women receiving care in emergency departments: implications for IPV and HIV screening. Emerg Med J 2007;24:255–9. 10.1136/emj.2006.041541 17384378 PMC2658230

[13] Mittal M, Senn TE, Carey MP Mediators of the relation between partner violence and sexual risk behavior among women attending a sexually transmitted disease clinic. Sex Transm Dis 2011;38:1–5. 10.1097/OLQ.0b013e318207f59b 21258269 PMC3106110

[14] Devine A, Spencer A, Eldridge S, et al Cost-effectiveness of Identification and referral to improve safety (IRIS), a domestic violence training and support programme for primary care: a modelling study based on a randomised controlled trial. BMJ Open 2012;2:e001008 10.1136/bmjopen-2012-001008 PMC3383977 22730555

[15] Yeung H, Chowdhury N, Malpass A, et al Responding to domestic violence in general practice: a qualitative study on perceptions and experiences. Int J Family Med 2012;2012:1–7. 10.1155/2012/960523 PMC3502852 23259062

[16] Pathak N, Sohal A, Feder GS How to enquire and respond to domestic violence and abuse in sexual health settings. Sex Transm Infect 2017;93:175–8. 10.1136/sextrans-2015-052408 27455852

[17] Sohal AH, Pathak N, Blake S, et al Improving the healthcare response to domestic violence and abuse in sexual health clinics: feasibility study of a training, support and referral intervention. Sex Transm Infect 2017:sextrans-2016-052866 10.1136/sextrans-2016-052866 PMC5870455 28724743

[18] Sandelowski M Sample size in qualitative research. Res Nurs Health 1995;18:179–83. 10.1002/nur.4770180211 7899572

[19] Boyatzis R Transforming qualitative information: thematic analysis and code development. CA, Sage: Thousand Oaks, 1998.

[20] Goicolea I, Vives-Cases C, Hurtig AK, et al Mechanisms that Trigger a Good Health-Care Response to Intimate Partner Violence in Spain. Combining Realist Evaluation and Qualitative Comparative Analysis Approaches. PLoS One 2015;10:e0135167 10.1371/journal.pone.0135167 26270816 PMC4536036

[21] Ritchie M, Nelson K, Wills R Family violence intervention within an emergency department: achieving change requires multifaceted processes to maximize safety. J Emerg Nurs 2009;35:97–104. 10.1016/j.jen.2008.05.004 19285170

[22] Robinson R Myths and stereotypes: how registered nurses screen for intimate partner violence. J Emerg Nurs 2010;36:572–6. 10.1016/j.jen.2009.09.008 21078473

[23] Williamson E, Jones SK, Ferrari G, et al Health professionals responding to men for safety (HERMES): feasibility of a general practice training intervention to improve the response to male patients who have experienced or perpetrated domestic violence and abuse. Prim Health Care Res Dev 2015;16:281–8. 10.1017/S1463423614000358 25248144

[24] Trevillion K, Howard LM, Morgan C, et al The response of mental health services to domestic violence: a qualitative study of service users' and professionals' experiences. J Am Psychiatr Nurses Assoc 2012;18:326–36. 10.1177/1078390312459747 22989418

[25] Drinkwater J, Stanley N, Szilassy E, et al Juggling confidentiality and safety: a qualitative study of how general practice clinicians document domestic violence in families with children. Br J Gen Pract 2017;67:e437–44. 10.3399/bjgp17X689353 28137783 PMC5442959

[26] Rees K, Zweigenthal V, Joyner K Implementing intimate partner violence care in a rural sub-district of South Africa: a qualitative evaluation. Glob Health Action 2014;7:24588 10.3402/gha.v7.24588 25226415 PMC4165045

[27] HM Government. Violence against women and girls Strategy 2016 - 2020. London: HM Government, 2016.

[28] Ford-Gilboe M, Wuest J, Varcoe C, et al Developing an evidence-based health advocacy intervention for women who have left an abusive partner. Can J Nurs Res 2006;38:147–67. 16671285

[29] Dowd MD, Kennedy C, Knapp JF, et al Mothers' and health care providers' perspectives on screening for intimate partner violence in a pediatric emergency department. Arch Pediatr Adolesc Med 2002;156:794–9. 10.1001/archpedi.156.8.794 12144370

[30] Hooker L, Small R, Humphreys C, et al Applying normalization process theory to understand implementation of a family violence screening and care model in maternal and child health nursing practice: a mixed method process evaluation of a randomised controlled trial. Implement Sci 2015;10:39 10.1186/s13012-015-0230-4 25890352 PMC4379540

[31] Brook G, Bacon L, Evans C, et al UK national guideline for consultations requiring sexual history taking. Clinical effectiveness group british association for sexual health and HIV. Int J STD AIDS 2014;25:391–404. 10.1177/0956462413512807 24285601

